# Targeted Therapy With Vemurafenib in Brazilian Children With Refractory Langerhans Cell Histiocytosis: Two Case Reports and Review of Literature

**DOI:** 10.1002/cnr2.2142

**Published:** 2024-08-27

**Authors:** Klerize Anecely de Souza Silva, Isis Maria Quezado Soares Magalhães, Daniela Elaine Roth Benincasa, Daiane Keller Cecconello, Mariana Bohns Michalowski

**Affiliations:** ^1^ Pós Graduação Ciências da Saúde da Criança e Adolescente Universidade Federal do Rio Grande do Sul Porto Alegre Rio Grande do Sul Brazil; ^2^ Hospital da Criança Conceição Porto Alegre Brazil; ^3^ Hospital da Criança de Brasília José Alencar Brasília Brazil

**Keywords:** BRAF mutations, Langerhans cell histiocytosis, vemurafenib

## Abstract

**Background:**

Langerhans cell histiocytosis (LCH) is a clonal myeloid neoplasm with inflammatory component. Refractory disease is a challenge, but vemurafenib has emerged as a therapeutic option. We will delineate the cases of two Brazilian children suffering from refractory LCH with a positive response to vemurafenib.

**Cases:**

Both cases had a diagnosis of multisystem disease with involvement of organs at risk and had not responded to standard and second‐line treatment. After refractoriness to classic treatment regimens, the BRAF mutation was investigated and found to be positive in both patients, and target therapy with vemurafenib was sought. The first case has been using vemurafenib for about 2 years and the second case has been using it for about 3 years, having had an attempt to suspend the medication after concomitant use with maintenance therapy. However, the disease returned 4 months after stopping the medication. Fortunately, the disease returned to remission status after the medication was reintroduced.

**Conclusion:**

These cases represent the first reported instances of off‐label vemurafenib use in Brazil for the treatment of LCH and both patients have demonstrated excellent responses to the medication. However, the long‐term side effects are unknown in children, and prospective studies are needed. In addition, there is a lack of epidemiological data on histiocytosis in Brazil and studies evaluating the budgetary impact of incorporating BRAF mutation research and the use of vemurafenib into the public health system. These reports could be a starting point.

## 1 Introduction

Langerhans cell histiocytosis (LCH) is a rare myeloid neoplasm characterized by inflammatory lesions featuring pathological clonal infiltration of cells belonging to the mononuclear phagocyte system, which exhibit phenotypical traits resembling Langerhans cells, notably expressing CD1a and CD207 [1]. It is an exceptionally rare disease, with reported incidence rates ranging from 2.6 to 8.9 cases per million among children under 15 years of age, peaking at an average age of approximately 3 years [2]. Notably, the disease presents a broad clinical spectrum, ranging from self‐limited forms to aggressively progressive multisystemic variants, often entailing an unfavorable prognosis [3].

To address this diversity, the therapeutic approach to LCH is intricately tailored based on the extent of organ involvement. In cases of localized, single‐organ disease, a watch‐and‐wait strategy may suffice. Alternatively, when isolated bone involvement occurs, surgical interventions may be considered. Notably, localized disease often carries a favorable prognosis [4].

Conversely, multisystemic disease is characterized by the presence of affected risk organs such as the bone marrow, spleen, and liver, significantly worsening the prognosis [5]. One persistent challenge in LCH treatment is the notable rate of disease reactivation, particularly in instances where risk organs are implicated [6]. Patients with refractory LCH typically exhibit characteristics such as age under 2 years, risk organ involvement, elevated inflammatory markers, and resistance to standard treatment and often harbor the BRAF V600E mutation [7].

Recent advancements have illuminated the pathophysiology of LCH, unveiling the pivotal role of the “Ras/Raf/MEK/ERK” signaling pathway in myeloid differentiation [8]. Within the spectrum of mutations, the BRAF mutation stands out as the most prevalent, accounting for over 50% of LCH cases. It is closely associated with severe clinical presentations, heightened resistance to conventional chemotherapy, and an increased risk of relapse [9]. The BRAF gene regulates the synthesis of the BRAF protein, a crucial component of the RAS/MAPK signaling pathway that governs fundamental cellular processes such as proliferation, differentiation, migration, and apoptosis [10]. In the presence of BRAF mutations, the aberrant BRAF protein perpetuates unregulated signaling to the cell nucleus, fueling unrestrained growth and differentiation of Langerhans cells [11]. Other genetic mutations, including those within the MAP2K and ARAF genes, have also been identified [12].

With these recent molecular insights into the central role of the BRAF mutation in LCH pathogenesis, vemurafenib has emerged as a therapeutic option for children grappling with high‐risk multisystemic disease that proves refractory to conventional treatments or experiences recurrent relapses. Vemurafenib, initially approved for metastatic melanoma in adults, has demonstrated the capacity to target and inhibit the BRAF V600E mutation [13]. While the off‐label use of vemurafenib in refractory multisystemic LCH among children persists in Brazil, the European Medicines Agency has sanctioned its utilization. Observational studies in Europe have already attested to the safety and efficacy of vemurafenib in children afflicted with refractory LCH carrying the BRAF V600E mutation [14]. However, it is crucial to underscore that this therapeutic approach has yet to definitively eradicate the neoplastic clone and long‐term toxicities remain a subject of ongoing investigation [15].

In this paper, we will delineate the cases of two Brazilian children suffering from refractory LCH, which remained unresponsive to both standard and second‐line treatments but exhibited a positive response to vemurafenib therapy. In both cases, written informed consent was obtained.

## 2 Case Report I

The patient is a 1‐year and 9‐month‐old female child who presented with pancytopenia and a history of atopic and seborrheic dermatitis, repeated febrile otitis, impetigo on the scalp, extensive skin lesions on the perineum and difficulty walking for 3 months. She was referred to a Brazilian tertiary hospital for investigation initially with suspicion of leukemia due to pancytopenia. Further examination revealed pale skin, lichenized skin lesions on the neck, erythematous and moist skin lesions on various body parts, including the perineum, as well as hepatosplenomegaly and suppurative otitis.

**FIGURE 1:**
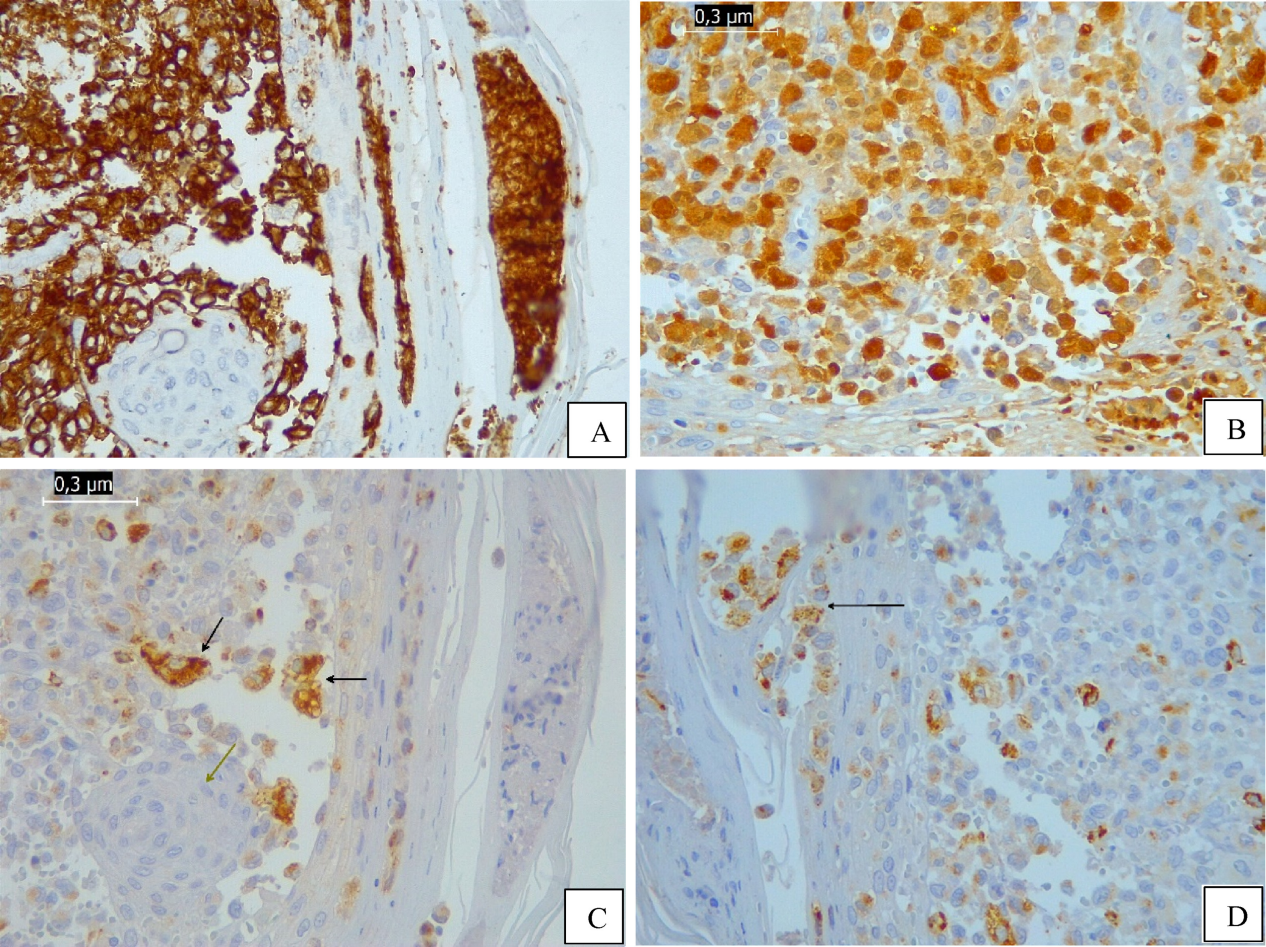
Immunohistochemistry (IHC) images of the skin biopsy at diagnosis of LCH. (A) IHC: Positive CD1a expression in the proliferated histiocytic cells; negative expression in the osteoclast‐type multinucleated cells. Magnification: ×400. (B) IHC: Positive S100 expression in the proliferated histiocytic cells. Magnification: ×400. (C) and (D) IHC: Positive CD68 expression in the proliferated histiocytic cells (black arrow); negative expression in the osteoclast‐type multinucleated cells (green arrow). Magnification: ×400.

CT scans showed numerous osteolytic lesions affecting multiple bones, including the skullcap, skull base, maxilla, mandible, scapulae, ribs, vertebrae, pelvis, and proximal femurs, in addition to hepatosplenomegaly. The patient underwent skin biopsy and bone marrow biopsy. Anatomopathology (AP) of the skin biopsy was compatible with LCH. Immunohistochemistry (IHC) of skin biopsy showed PS 100 (anti‐human S100): positive; CD1a (clone EP3622): positive; CD68 (clone Kp1): positive (Figure 1). Bone marrow showed infiltration by LCH.

After examination results confirmed the clinical suspicion of Multisystemic LCH (MS—LCH) with involvement of risk organs (liver, spleen, and bone marrow) and bone involvement. The patient started chemotherapy treatment for Multisystemic Histiocytosis—Langerhans Cell Histiocytosis Protocol (LCH 2009) with vinblastine 6 mg/m^2^ weekly + prednisolone 40 mg/m^2^ daily. After 6 weeks of treatment referring to cycle 1, reevaluation examinations were performed, which showed practically no improvement. She received cycle 2 of chemotherapy treatment with vinblastine and prednisone for another 6 weeks according to protocol. After the first 2 cycles of treatment, the child was in better general condition, skin looked a little better, nodules on the skull cap were no longer palpable, suppurative otitis resolved, but still with a large volume of disease. Persisted with hepatosplenomegaly, compromised bone marrow, and multiple bone lesions picking up on bone scintigraphy. Impression of disease refractory to treatment.

Opted for treatment switch to second‐line therapy regimen with cladribine 5 mg/m^2^ D1—D5+ cytarabine 100 mg/m^2^ D1—D4. After 2 cycles of treatment persisted with bone marrow infiltration by LCH. Throughout the chemotherapy treatment, the patient had several serious infectious complications such as febrile neutropenia, sepsis, central catheter infections, typhlitis, and the need to be admitted to the pediatric intensive care unit for management of severe complications with risk of death. After the sixth cycle of second‐line chemotherapy, she was reassessed. The skin lesions had a complete response. The girl is walking again with no signs of pain. However, she had a partial response to second‐line treatment, the disease proved refractory to treatment due to the persistence of hepatosplenomegaly, osteolytic lesions in bones of the face, skull cap and skull base, and spinal cord involvement and persisted with bone marrow infiltration by histiocytes (IHC compatible with the residual presence of LCH [PS100 (anti‐human S‐100): positive, focal; CD1a (clone EP3622): positive, focal; CD68 (clone KP1): positive focal]).

BRAF V600E mutation testing was performed on a skin biopsy sample from the diagnosis, which showed the presence of the V600E variant in the BRAF gene. The child had no other chemotherapy treatment option available through the Brazilian Unified Health System. The medication vemurafenib (zelboraf) was then requested through the courts. She started treatment at the recommended dose for children of 10 mg/kg twice a day and has been using it for 2 years and 4 months.

**FIGURE 2:**
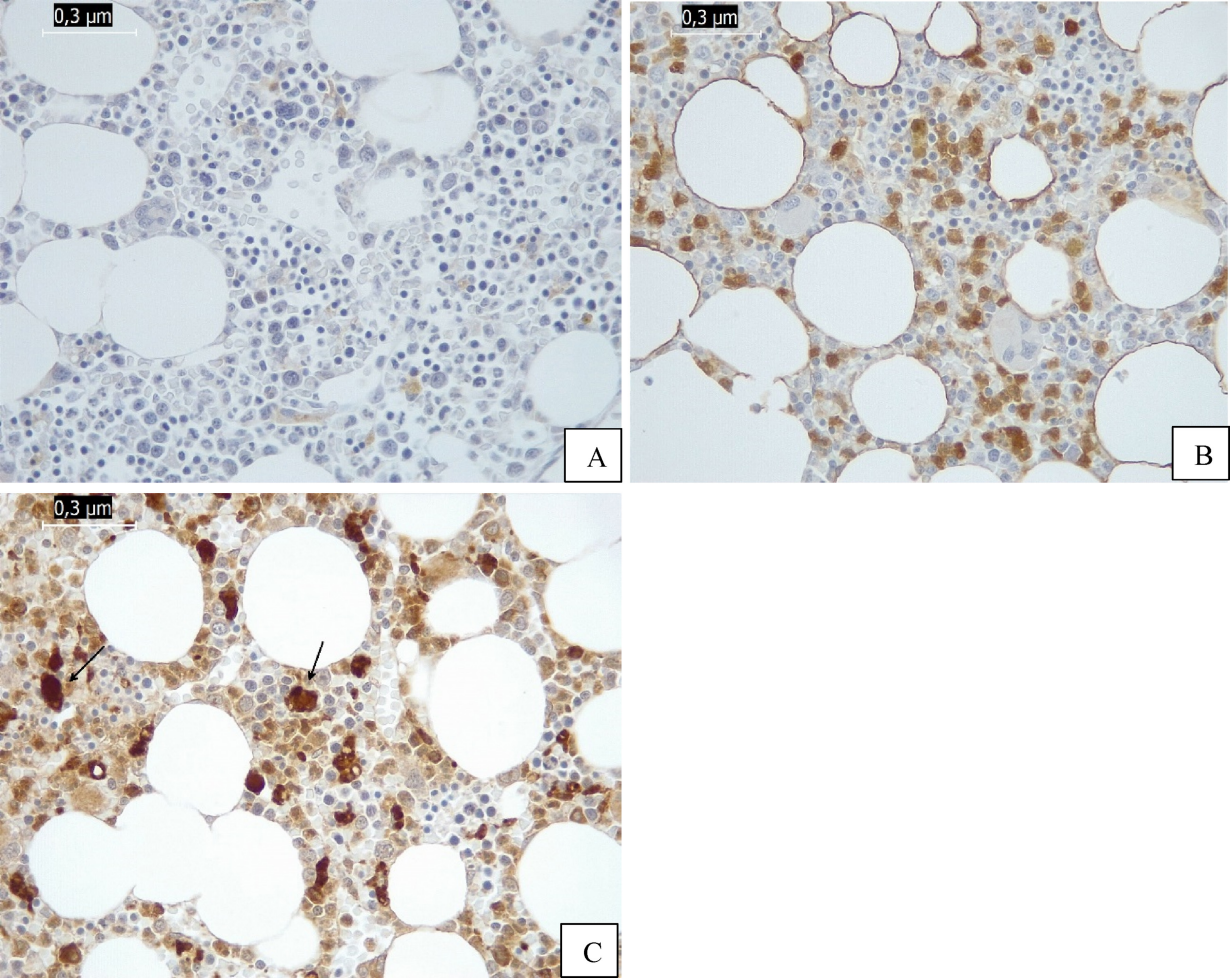
Immunohistochemistry (IHC): Images of the negative bone marrow after treatment with vemurafenib. (A) IHC: Negative CD1a expression. Absence of Langerhans cells. Magnification: ×400. (B) IHC: Negative S100 expression. Positive in macrophages. Magnification: ×400. (C) IHC: Positive CD68 only expression in hemosiderophages. Magnification: ×400.

**FIGURE 3:**
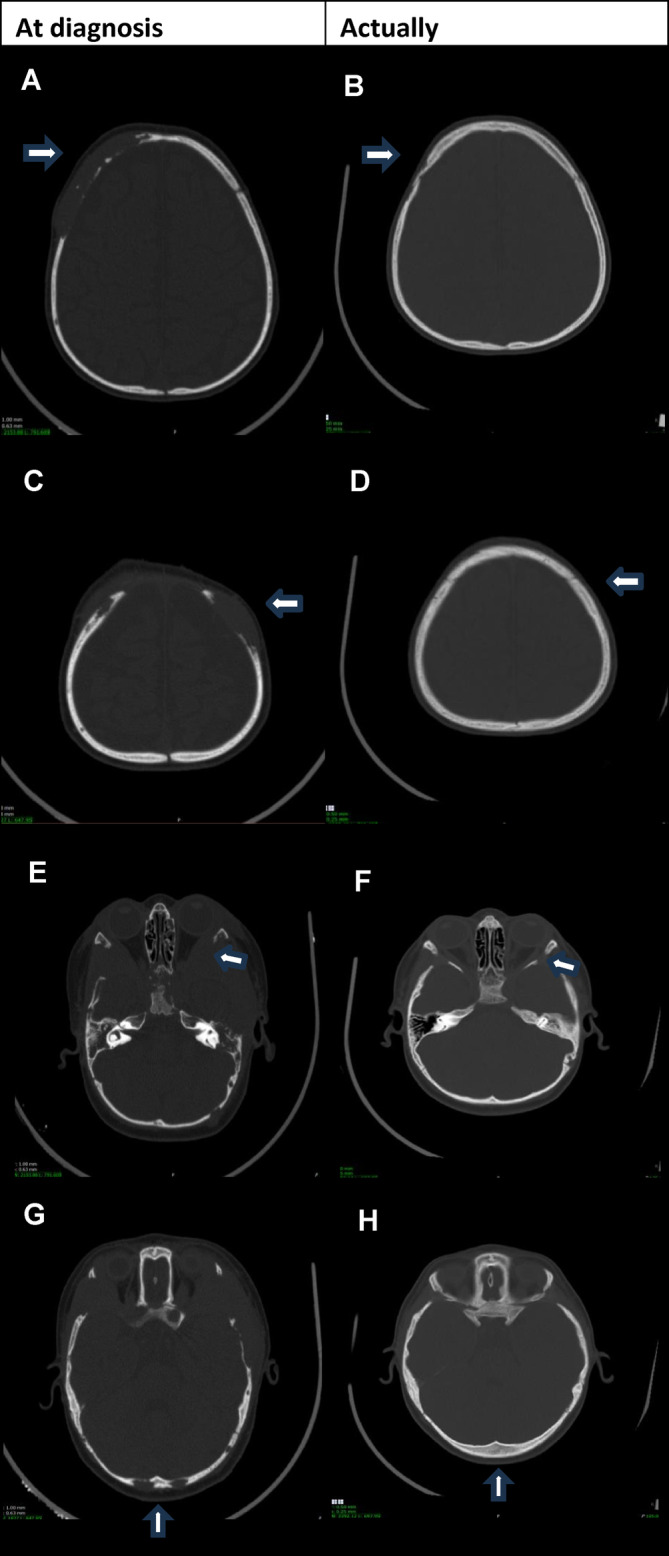
Images from CT scans of the skull that compare the presentation of bone lesions in the skullcap and skull base at the time of diagnosis with the current images (2 years of vemurafenib). (A) Right upper skullcap (extensive osteolytic lesion with expansive soft tissue component). (B) Right upper skullcap (significantly smaller radiolucent bone area). (C) Left upper skullcap (osteolytic lesion with expansive soft tissue component). (D) Left upper skullcap (osteolytic lesion no longer individualizable). (E) Skull base/lateral orbital wall on the left (extensive osteolytic lesion with expansive soft tissue component). (F) Skull base/lateral orbital wall on the left (significantly smaller radiolucent bone area). (G) Bilateral occipital (osteolytic lesions with expansive soft tissue component). (H) Bilateral occipital osteolytic lesions (no longer individualizable).

The patient was reassessed after 7 months of medication and the bone marrow for the first time showed no infiltration by LCH (Figure 2). At present, there is only stable residual lytic lesions in the left mastoid, skull base, and left mandible, with areas of ossification/remodeling appearing around the skull base lesions. Figure 3 shows images from CT scans of the skull that compare the presentation of bone lesions in the skullcap and skull base at the time of diagnosis with the current images of these regions, showing significant improvement.

The patient currently takes vemurafenib 6.8 mg/kg/dose every 12 h, without significant side effects, maintaining an excellent quality of life at 5 years and 5 months of age.

## 3 Case Report II

**FIGURE 4:**
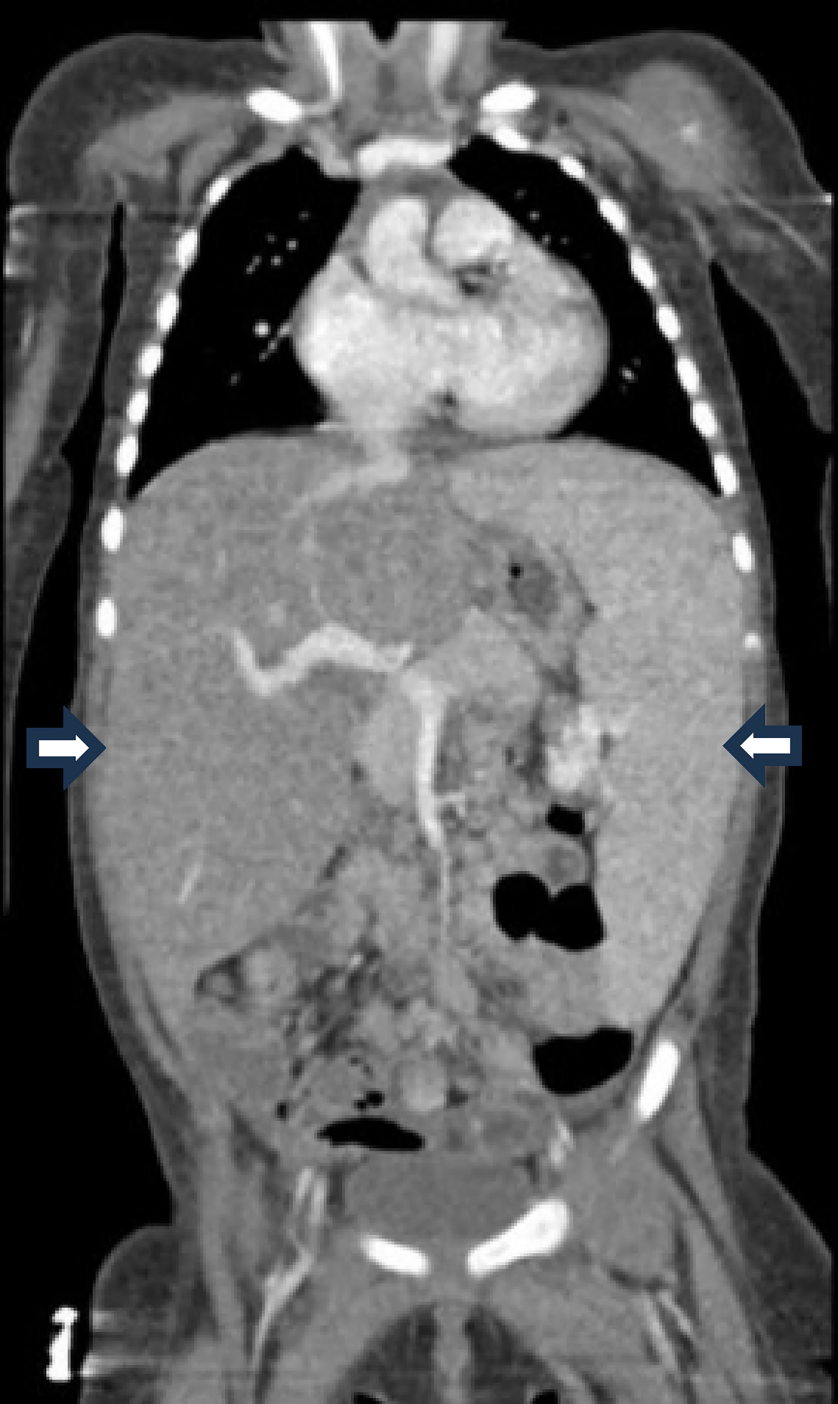
Hepatosplenomegaly visualized on abdominal computed tomography.

The patient is a female child, 1 year and 1 month old, with a history of recurrent otorrhea, hospitalizations for otitis, diarrhea, and dehydration. During a hospitalization for inappetence and diarrhea, pancytopenia and hepatosplenomegaly were observed, leading to a referral to a Brazilian pediatric oncology service. Physical examination revealed hepatosplenomegaly, a tumor in the occipital region, skin abnormalities including petechiae on the thighs and inguinal region, and dermatitis on the scalp. Figure 4 demonstrates hepatosplenomegaly visualized on abdominal computed tomography.

**FIGURE 5:**
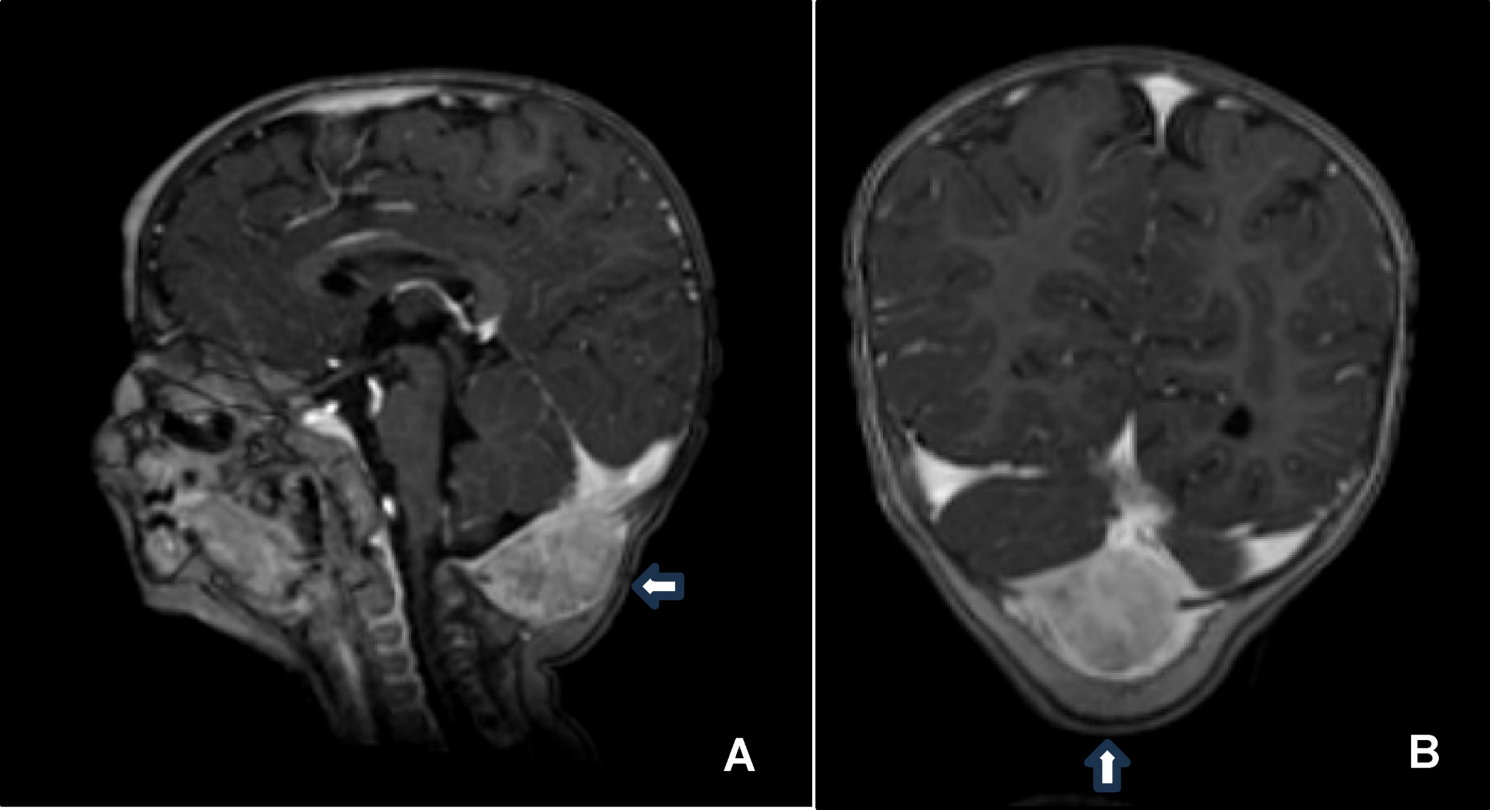
(A) and (B) Magnetic resonance imaging with postcontrast T1‐weighted sequence showing a locally aggressive expansive bone lesion in the occipital region, with evident enhancement and proximity to the torcula. There is no compressive effect on the structures of the posterior fossa.

Imaging confirmed a voluminous solid expansive formation in soft tissue density involving the occipital region, exerting a mass effect on the posterior fossa and surface of the scalp with osteolytic behavior on adjacent structures measuring approximately 3.8 × 3.9 × 4.1 cm (Figure 5).

**FIGURE 6:**
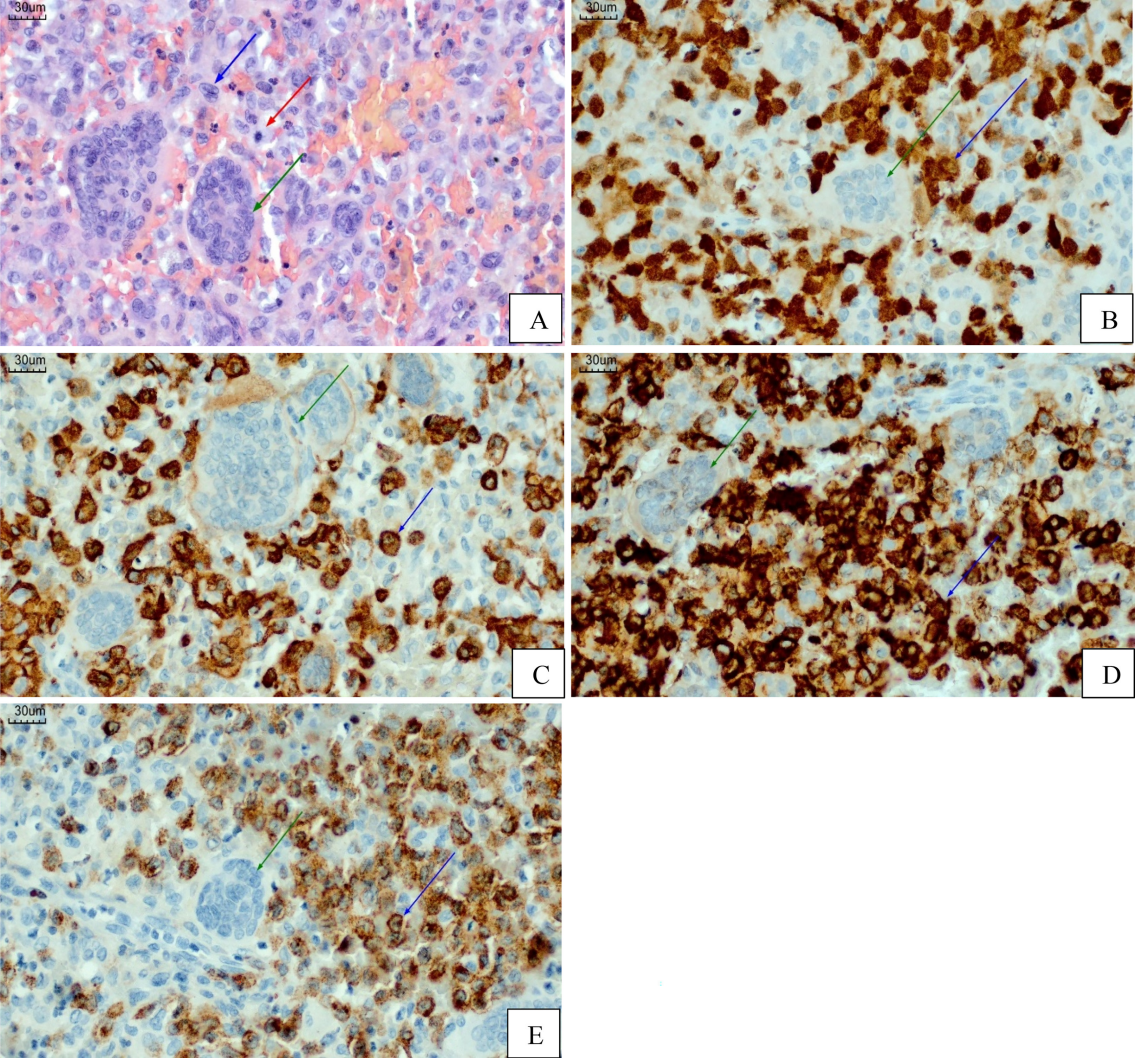
(A) Photomicrography of a slide stained with hematoxylin and eosin (HE) of the occipital lesion showing its histopathological features: Proliferation of large, rounded/oval histiocytic cells with complex nuclear contours, frequent nuclear grooves, convoluted nuclei, clear, vesicular chromatin, and moderate amount of light, eosinophilic cytoplasm (blue arrow). Many typical mitotic figures are seen (red arrow). In the background, there is an inflammatory microenvironment made up of eosinophils, lymphocytes, and some multinucleated cells (osteoclast‐type). Scale bar: 30,0 μm. Magnification: ×400. (B) Immunohistochemistry (IHC): Positive S100 expression (nuclear, cytoplasmatic and membrane) in the proliferated histiocytic cells (blue arrow); negative expression in the osteoclast‐type multinucleated cells (green arrow). Scale bar: 30,0 μm. Magnification: ×400. (C) IHC: Positive CD163 expression (cytoplasmatic and membrane) in the proliferated histiocytic cells (blue arrow); negative expression in the osteoclast‐type multinucleated cells (green arrow). Scale bar: 30,0 μm. Magnification: ×400. (D) IHC: Positive CD1a expression (cytoplasmatic and membrane) in the proliferated histiocytic cells (blue arrow); negative expression in the osteoclast‐type multinucleated cells (green arrow). Scale bar: 30,0 μm. Magnification: ×400. (E) IHC: Positive CD207 (langerin) expression (cytoplasmatic) in the proliferated histiocytic cells (blue arrow); negative expression in the osteoclast‐type multinucleated cells (green arrow). Scale bar: 30,0 μm. Magnification: ×400.

The histopathological report and IHC of the occipital lesion biopsy were compatible with LCH. IHC showed CD1a (clone O10): positive; Protein S100 (polyclonal): positive; CD207 (clone 12D6): positive; CD163 (clone CD163): positive (Figure 6).

Chemotherapy was initiated according to the LCH 2009 protocol with vinblastine 6 mg/m^2^ weekly + prednisolone 40 mg/m^2^/day (multisystemic staging = skin, hematopoietic, bone, and hepatosplenomegaly) with an initial partial response of skin lesions and spleen and liver volume. After Week 7 of treatment, noted clear recrudescence of skin lesions on scalp and hepatosplenomegaly, defined refractoriness, and opted for second‐line therapy (cytarabine 33 mg/kg/day of 12/12 h in intravenous infusions of 2 h + cladribine 0.3 mg/kg for 5 days). Second‐line therapy with cytarabine and cladribine was attempted but had to be suspended in the second cycle due to septic shock.

A BRAF mutation test confirmed the p.V600E mutation in exon 15 of the BRAF gene on occipital bone lesion biopsy, leading to the initiation of vemurafenib therapy. Initially, vemurafenib showed a good response, with a reduction in organomegaly and resolution of skin lesions. After 12 months of continuous vemurafenib therapy, maintenance antineoplastic therapy was introduced according to the LCH 2009 protocol (prednisolone 40 mg/m^2^/day for 5 days every 3 weeks + vinblastine 6 mg/m^2^ every 3 weeks + mercaptopurine 50 mg/m^2^ continuously). She used this association for 12 months, when vemurafenib and chemotherapy were suspended. However, skin lesions recurred after 4 months of discontinuing vemurafenib. The medication was reintroduced without adapting the dose to weight, and the patient returned to the previous remission status. She is currently taking a dose of 5.8 mg/kg/dose every 12 h and has been on vemurafenib for a total of 36 months, maintaining complete remission, excellent overall health, and quality of life.

The patient is currently 5 years and 3 months old and continues to use vemurafenib as the sole treatment.

## 4 Discussion

Histiocytosis encompasses a diverse range of diseases classified as inflammatory myeloid neoplasms, stemming from common myeloid progenitors. These diseases span various cells of the mononuclear phagocytic system and are further categorized into dendritic cell (DC) disorders, macrophage‐related disorders, and malignant histiocytic disorders [16]. Historically, the classification of histiocytic cell disorders was primarily based on the presence of Langerhans cells in tissue, identified by their positivity for CD1a and CD207 (Langerin) [17]. Among these disorders, LCH stands out as the most prevalent in humans. Initially considered a reactive disorder, it was later confirmed that the Langerhans cell histiocytes found in lesion sites are clonal in nature [18].

The discovery of the recurrent BRAF gene mutation's involvement in histiocytosis pathogenesis is relatively recent. In 2010, Badalian‐Very et al. first described this mutation, suggesting that histiocytosis is a neoplastic disease driven by the BRAF V600E gain‐of‐function mutation. This mutation is found in approximately 57% of histiocytosis cases and is a key discovery given the disease's heterogeneity [19]. Importantly, this mutation leads to constitutive activation of the downstream kinases, MEK, and extracellular signal‐regulated kinase (ERK) and is not unique to histiocytosis. It is a common oncogenic mutation observed in about 30 different neoplasms (e.g., melanoma, hairy cell leukemia, and thyroid carcinoma) as well as many benign conditions such as melanocytic nevi and colon polyps [20].

Over the past decade, mutually exclusive activated MAPK pathway genes have been identified in over 85% of LCH lesions, a finding that is in line with universal ERK activation observed in LCH cells [21]. In addition to BRAF‐V600E, alternative mutations include tyrosine kinase receptors (e.g., ERBB3), BRAF (fusions, deletions, duplications), ARAF, and MAP2K1 (encodes MEK). Mutations in MAP2K1 have been found in 50% of BRAF wild‐type patients, indicating the critical role of the MAPK pathway in LCH pathogenesis. This finding may have implications for the use of therapy not only with BRAF inhibitors, but MEK inhibitors as well [22]. Other mutations affecting ARAF, MAP3K1, NRAS, PI3CA, and other targets are found in the remaining 25% of patients [23].

Peripheral blood mononuclear cells (PBMCs) harboring the BRAF‐V600E mutation have been detected in children with active high‐risk clinical LCH, particularly those with liver, bone marrow, and spleen involvement, although occurrences in low‐risk LCH cases are rare. Additionally, hematopoietic cell progenitors (CD34+) carrying the BRAF‐V600E mutation have been identified in patients with high‐risk LCH. Notably, enforced expression of BRAF‐V600E in myeloid precursors in a mouse model has been shown to induce LCH‐like disease [24]. These collective findings lend support to the theory of dysregulated myeloid DCs in LCH pathogenesis, with high‐risk LCH stemming from pathological activation of ERK in hematopoietic progenitor cells, while low‐risk disease arises from ERK activation in tissue‐restricted precursors. Consequently, LCH is currently recognized as an inflammatory neoplastic disorder [25].

Regarding immune dysfunctions, LCH lesion CD207+ DCs exhibit elevated levels of T‐cell costimulatory molecules and proinflammatory cytokines. Activation of MAPK in myeloid precursors disrupts CCR7 expression, leading to the entrapment of activated LCH cells within lesions [26]. Multiple cytokines, chemokines, and their receptors have been implicated in LCH pathogenesis. Furthermore, LCH cells express programmed death‐ligand 1 (PD‐L1), an immune checkpoint regulator. However, the mechanisms by which pathological cells in LCH lesions incite inflammation and the roles of recruited T cells in the disease process remain incompletely understood [27].

For childhood LCH necessitating systemic therapy, the first‐line treatment typically involves a combination of vinblastine and corticosteroids. However, approximately 50% of LCH patients either do not respond to first‐line induction chemotherapy or experience disease reactivation within the initial 5 years of diagnosis [28]. Determining the optimal treatment for relapsed or refractory LCH remains a topic of debate and should be tailored based on the initial disease extent and risk organ involvement. Treatment options include cladribine monotherapy, cladribine with cytarabine, clofarabine, MAPK pathway inhibitors (BRAF or MEK inhibitors), and, less frequently, hematopoietic stem cell transplant (HSCT) [29].

Currently, it is recognized that the BRAF V600E mutation in LCH is associated with an increased risk of treatment failure, as well as LCH‐associated neurodegeneration [30]. In this context, the use of the selective BRAF inhibitor, vemurafenib, has been used to treat refractory LCH [31]. Initial reports, including the case by Heritier et al., demonstrated the successful use of vemurafenib in children with resistant MS‐LCH [32]. Most studies and case reports describe vemurafenib use in relapsed or refractory histiocytosis cases that did not respond to first and/or second‐line treatment. These cases often involve severely ill infants who showed rapid responses to vemurafenib and vemurafenib has proven to be a safe treatment with no serious adverse effects during its use [33].

Prior to targeted therapy, second‐line treatments for histiocytosis were associated with high toxicity, making them less favorable. One of the known regimens constitutes higher dose cytarabine plus standard dose cladribine, also known as 2′‐chlorodeoxyadenosine or 2‐CdA. This regimen demonstrated an overall 5‐year survival of 85% but was associated with high toxicity [34]. HSCT was considered a rescue option, but its toxicity was even higher. Kudo et al. showed overall survival (OS) of 57% of patients with refractory LCH who underwent HSCT, but OS correlated with disease status [35].

It is worth noting that BRAF or MAPK inhibitors block the differentiation and proliferation of mutant cells, but do not eradicate the mutant clone in the blood and bone marrow [36]. To try to overcome this, some studies have chosen to combine vemurafenib with chemotherapy. A single‐center retrospective study of 15 patients with BRAF V600E mutation and LCH showed that conventional doses of cytarabine with lower doses of 2‐CdA appeared to be unable to eradicate LCH progenitor cells. All patients had previously received various chemotherapy regimens for LCH and first‐line therapy for all patients was vimblastine and prednisolone for more than 6 weeks. Seven patients received at least one course of treatment using citarabine plus 2‐CdA and one patient received monotherapy using 2‐CdA. The median duration of vemurafenib treatment was 29 months from the start of medication and only one patient was able to discontinue vemurafenib treatment without reactivation. They did not find a reasonable explanation for this unique success, but further studies using new technologies for monitoring minimal residual disease (MRD) could shed light on this issue [15].

Another retrospective study of 17 pediatric patients was evaluated the efficacy and safety of vemurafenib combined with low‐toxicity chemotherapy in patients with severe or refractory LCH. Its data suggested that the combination of vemurafenib and chemotherapy can achieve sustained clinical and molecular‐level relief in children with LCH. Nevertheless, the optimal combination strategy remains unclear [37].

In relapsed and/or refractory LCH, vemurafenib alone induces a complete response in 70% or a partial response in 30% of children, with less toxicity than second‐line chemotherapy such as cladribine and cytarabine or allogeneic bone marrow transplantation. However, discontinuation of vemurafenib results in complete relapses that cannot be prevented even with the use of concomitant chemotherapy [38].

These findings are in agreement with a cohort of 54 patients coordinated by Donadieu et al. In this cohort, patients had refractory multisystemic histiocytosis and were treated with vemurafenib at a dose of 20 mg/kg/day with a mean treatment duration of 13.9 months and a range of 2–38 months. The BRAF V600E mutation load in circulating cells decreased, but at no time did it become negative, indicating a high risk of relapse after cessation of vemurafenib. The results of this cohort suggest that although vemurafenib was highly effective in stabilizing the disease, it unfortunately does not cure the disease [14].

However, a major concern with long‐term vemurafenib treatment is its potential for toxicity, as observed in adult patients with Erdheim‐Chester disease. In these patients, the most common side effects were skin complications, ranging from pilar keratosis and photosensitivity to spinocellular carcinoma and melanoma [39]. In a meta‐analysis including studies with adults and children, the most common adverse events were also cutaneous [40]. In another study whose objective was to evaluate cutaneous adverse events in children, it was shown that they are rarely serious and have little impact on the continuation of treatment when managed appropriately [41]. Therefore, vemurafenib's safety profile appears more favorable in infants and children, although long‐term effects remain unknown [15]. In the case reports described, the two children have not shown any side effects to the medication so far.

In addition to vemurafenib, there are other BRAF inhibitors that can be used in BRAF‐mutant LCH such as dabrafenib and other MAPK inhibitors such as trametinib and cometinib that are broader spectrum and can be used in LCH with alterations in other parts of the MAPK pathway, even those involving more downstream signaling molecules. However, there is a lack of data for the use of these other inhibitors in children [42].

Finally, vemurafenib appears as a highly effective and safe therapeutic option in the treatment of BRAF mutated refractory LCH. However, the timing of initiation, dose, and duration of therapy are not well established. Collaborative prospective trials are required to determine optimal dose and duration of vemurafenib for patients with LCH and to compare it with alternative MAPK inhibitors, combination therapies or HSCT [40].

Furthermore, when it comes to children, the questions increase. Although vemurafenib appears to be safe and effective in pediatric patients with refractory or relapsed LCH associated with BRAF mutation, the data available in the literature are still limited, as most are based on case reports or case series. Additionally, Brazil lacks epidemiological data on histiocytosis and studies evaluating the budgetary impact of incorporating BRAF mutation research and the use of vemurafenib into the public health care system.

While the expense associated with vemurafenib treatment can be significant, it is essential to consider that this is a rare disease that primarily affects young children. Historically, high‐risk refractory cases have had low survival rates, making remission‐inducing treatment invaluable. Targeted therapy has the potential to extend and enhance the quality of life for patients and their families. These factors should not be underestimated, as they encompass the reintegration of the child and their family into society. Nevertheless, it is crucial to acknowledge that further data are required to firmly solidify these conclusions.

## 5 Conclusion

The cases presented here represent the first reported instances of off‐label vemurafenib use in Brazil for the treatment of high‐risk LCH refractory to first‐ and second‐line therapies and both cases have shown excellent responses to the medication. Initially, they started taking a dose of 10 mg/kg/dose every 12 h. It was decided to maintain the initial dose without adjusting weight over time and both children remain in complete remission and did not present any adverse effects associated with the medication until now. However, the long‐term safety and efficacy of vemurafenib in children with LCH require further investigation.

The description of these cases emphasizes the importance of always seeking the best treatment for our patients, even with the financial limitations that exist in low‐income countries like Brazil. Furthermore, although there is a lack of objective data for a series of questions for pediatric use, we have proven data that vemurafenib acts as an inhibitor of the BRAF V600E mutation. Therefore, we need to seek formal release and approval of the use of this medication by health regulatory agencies for use in pediatric patients when indicated. For this, prospective studies in children are necessary and essential to address critical aspects, including histiocytosis pathophysiology, the sufficiency of vemurafenib as a standalone treatment in cases with BRAF mutations, timing of initiation, dose ideal, duration and the consideration of targeted therapy as a chronic treatment option.

## Author Contributions

**Klerize Anecely de Souza Silva:** conceptualization, investigation, methodology, writing – original draft, formal analysis, writing – review and editing. **Isis Maria Quezado Soares Magalhães:** investigation, writing – original draft, methodology, formal analysis. **Daniela Elaine Roth Benincasa:** writing – review and editing, investigation. **Daiane Keller Cecconello:** investigation, writing – review and editing. **Mariana Bohns Michalowski:** conceptualization, investigation, writing – original draft, writing – review and editing, methodology.

## Conflicts of Interest

The authors declare no conflicts of interest.

## Data Availability

The data that support the findings of this study are available on request from the corresponding author. The data are not publicly available due to privacy or ethical restrictions.
